# Social Network Gap Analysis Evaluation: A Case Study of the Southeastern Health Equity Council

**DOI:** 10.1097/FCH.0000000000000210

**Published:** 2018-11-21

**Authors:** Candace Forbes Bright, Thometta Cozart, Braden Bagley, Hannah Scott, Jonathan Dennis

**Affiliations:** Department of Sociology & Anthropology, East Tennessee State University, Johnson City (Dr Bright); Department of Health Sciences, The University of Alabama, Tuscaloosa (Ms Cozart); School of Communication (Mr Bagley) and Department of Political Science, International Affairs, and International Development (Mr Dennis), The University of Southern Mississippi, Hattiesburg; and Department of Public Health, Jackson State University, Jackson, Mississippi (Ms Scott).

**Keywords:** health coalitions, partnerships, social network gap analysis, Southeastern Health Equity Council

## Abstract

Despite the growing emphasis on collaboration in public health, there remains a dearth of literature providing tools for the evaluation of coalitions and councils. This study employed social network gap analysis as an evaluation tool. Survey data collected from the Southeastern Health Equity Council members were used to assess connections among members as a whole, by committee, by state, and by health specialty area. Analysis of how well Southeastern Health Equity Council met the representation outlined in its strategic plan was also conducted. Recommendations for improving the network and opportunities to effectively recruit and advance the work of Southeastern Health Equity Council are discussed.

DESPITE wide-reaching efforts to address health disparities, health outcomes exhibit persistent and widening gaps.^1^,^2^ In terms of health disparities, place matters. Populations in the southeast United States are disproportionately impacted in nearly all health indicators, including obesity, human immunodeficiency virus, infant mortality, violence, and numerous chronic diseases. Within these populations, further disparities exist by race, ethnicity, gender, sexual orientation, gender identity, and disability status.^3^

The complexity of these health disparities has resulted in the deeper specialization of individuals and organizations working to address community health, minority health, and health disparities.^4^ This deeper specialization introduces greater complexity and diversity to issues surrounding health disparities but in many cases, also results in discrete segregated efforts. In response, public health efforts seek to span these diverse specialties and efforts through coalition building.^4^ Through collaboration across multiple partners, coalition building has numerous theoretical advantages, such as the formation and promotion of relationships across organizations otherwise working parallel toward a common public health goal.^5^,^6^ Moreover, effective collaboration brings together diverse actors for problem-solving purposes and presents opportunities to improve the performance of each actor.^6^

It is widely accepted that partnerships and collaboration among diverse individuals with unique contributions are imperative for achieving public health goals.^7^,^8^ As such, public health initiatives often involve the establishment and use of cross-sector partnerships. As many diverse partners are needed to meet the needs of public health goals of reducing disparities, the partnerships among these individuals is of interest from an evaluation standpoint. According to Schoen et al,^7^^(p90)^

Integrated efforts to address public health issues by involving multiple stakeholders are expected to result in better health outcomes than programs not using a network approach. The rationale behind this is that no single organization has full control over all of the determinants of population health.

Based on this approach, public health councils and coalitions (herein councils) are formed as an integrated approach that pools knowledge and resources for the purpose of impacting the health of a targeted population.^9^ Despite the popularity of forming public health councils, there remains a lack of substantive empirical research on council evaluation. This lack of evaluation of the structure and collaboration of councils is largely due to the fact that it is challenging.^7^,^10^ Despite a growing emphasis on approaches around partnerships, these partnerships are often assumed and rarely measu-red^11^ or evaluated.^5^ Therefore, we need tools to inscribe accountability to this approach in order to advance the science of health disparities and health equity and “to achieve the highest level of health for all people.”^12^^(par 5)^

Social networks comprise relationships between individuals or organizations. To capture these relationships, social network analysis—herein defined as the systematic analysis of relationships among a bounded group of individuals^13^—provides techniques and tools, as well as theories, for understanding interaction.^14^ Social network analysis is used to measure the formation, structure, and progression of relationships, and provides tools to help evaluate coalitions by showing their structure and the processes among members.^5^,^15^–^17^ To conduct social network analysis research, data are collected and recorded on who is connected to whom and often at what level of relationship (ie, how well actors know each other) these connections occur. These data are then used to derive network measures to answer a research question. According to Valente et al,^14^ social network analysis is especially relevant for understanding, guiding, and improving relational processes. More specifically, social network analysis techniques can be used to better understand collaboration networks as this approach helps recognize the potential pitfalls of collaboration and find possible remedies.^18^–^20^

The use of social network theory—the theory that social network structures influence social dynamics and interactions^21^—and associated analyses has expanded immensely in public health in the past decade.^14^ Social network analysis in public health has classically focused on the interpersonal transmission of infectious diseases and more recently has been extensively applied to assess behaviors associated with chronic conditions.^14^,^22^ Among the many applications of social network analysis, it can be used to measure partnership characteristics to evaluate collaboration and the effectiveness of partnerships.^5^,^7^,^16^

One approach to social network evaluation of councils is to use network metrics to assess potential “gaps,” herein defined as aspects of network structure that might limit the effectiveness of the council. Gap analysis was first introduced in business process and service quality improvement research by Parasuraman et al^23^ to assess gaps of perception between actual and expected customer experiences. Within business studies, gap analysis has evolved to become the assessment of the impact of the process on the outcomes.^24^ In the context of public health collaboration, there is a need to better understand the network as a process preceding the advancement of their efforts. Utilizing social network analysis tools within gap analysis research allows us to concentrate on evidence-based change in council development. This research highlights the utility of social network gap analysis (SNGA) by using social network analysis tools to describe the relationships among the members of a council to reveal differences in communication and collaboration, and these data are used to inform council capacity and assess gaps.

Traditional and current measures of coalition effectiveness lack the ability to assess the structure of the network. Council effectiveness is often measured by member satisfaction, commitment to the coalition, and the quality of planning efforts.^25^ While studies have also assessed trust,^26^ inclusiveness,^27^ and effectiveness^28^ among members, there are to date no published studies using social network analysis for conducting a partnership gap analysis and associated partnership database. To highlight the utility of SNGA, this research presents a case study of the Southeastern Health Equity Council (SHEC).

Formed in 2011, SHEC is one of 10 regional health equity councils under the National Partnership for Action to End Health Disparities (NPA). Spearheaded by the US Department of Health & Human Services Office of Minority Health, NPA is the first national multisector community- and partnership-driven effort on behalf of health equity. The SHEC corresponds to US Department of Health & Human Services Region IV (RHECIV), which comprises 8 states in the American Southeast: Alabama, Florida, Georgia, Kentucky, Mississippi, North Carolina, South Carolina, and Tennessee. This voluntary association of 40 voting members (5 per state) is designed to bring together leaders from diverse backgrounds in minority health and health disparity elimination, including health care providers, health care–focused organizations, academia, public health agencies, economic development, faith-based organizations, grassroots organizations, and other nonprofit organizations and businesses. The diversity of SHEC ensures adequate input from diverse sectors on the council's efforts to understand and address health disparities in the region. According to the SHEC strategic plan, the stated purpose of SHEC is “to build collaboration and partnerships to achieve health equity in the Southeast region of the US” with the mission “to inform policy, change systems, and increase awareness for health equity ... in the target areas of health care access, healthy food access, cultural competence, and health disparities knowledge and awareness.” Also stated in the strategic plan, SHEC, like many health councils, is designed to bring together members from broad areas (both geographic and expertise in health) to form partnerships deemed necessary to address health disparities.

One area in which collaboration can be enhanced is through the systematic study of the relationships present and the opportunity for growth and change within council networks. Using SHEC as a case study, this research introduces SNGA methods for public health collaboration and partnerships. The SNGA is herein defined as an assessment of a network for the purpose of identifying the difference between the actual and potential performance of said network. More specifically, we present SNGA as a method for evaluating networks, such as councils, to assess the existence of relationships for the purpose of making recommendations for improvement to those relationships and the network more broadly.

The purpose of this article is to use SHEC as a case study to highlight the utility of SNGA for improving council effectiveness. In doing so, we (1) contribute to SHEC's strategy to address health disparities in the region through the gap analysis of partnerships, (2) develop a database of SHEC partnerships, and (3) make recommendations to SHEC on how to improve and utilize existing partnerships. In examining the existing collaborative relationships among members of SHEC, we make recommendations for improving the ability of the Council's effectiveness in meeting its stated goals. Moreover, through documenting the processes used in this case study, we contribute to the literature on collaboration evaluation through the introduction of SNGA.

## METHODS

To collect the data needed to assess partnerships among the members of SHEC, we conducted an online survey to solicit information on the relationships and affiliations for each member. The purpose of the survey was to develop social networking models in an effort to better understand partnerships, reach the desired goal of analyzing partnerships among SHEC, and develop a better understanding of the broad-based constituency served by the Council for the purposes of improving collaborative partnerships and ultimately advancing regional efforts around health disparities.

### Survey development

A survey instrument was adapted from the one used for the evaluation of the Gulf States Health Policy Center coalition^5^ and modeled after the PARTNER Tool developed by the University of Colorado Center on Network Science.^29^ The study and survey were approved by the Institutional Review Board at The University of Southern Mississippi and solicit information from SHEC members on their primary organization, their affiliations, their state and committee representation on SHEC, and their relationship with all other SHEC members.

### Distribution and recruitment

The survey was distributed by e-mail and administered through Survey Monkey to all active SHEC members as of May 2017. The survey was open for 4 weeks. In partnership with a Health Equity Internship Program, funded by the Centers for Disease Control and Prevention (cooperative agreement no. 6U38OT0001370302), a master's-level student served as a 12-week intern and sent reminder e-mails to all members who had not taken the survey after week 1, as well as reminder phone calls as needed after weeks 2 and 3.

### Data analysis

The data were analyzed by first looking at descriptive statistics of the nonsocial network questions. We calculated frequencies of responses by the sector of the respondent's primary position (ie, academic in public health, academic not in public health, nonprofit with a primary focus in public health, nonprofit with primary focus other than public health, local government, state government working in public health, state government not working in public health, health care professionals, faith-based organization, media or communication, private for-profit business, or other) and by the committee on which they served (cultural competency, social determinants of health, governance, health care access, and violence as a public health issue). To evaluate the perception of Council's effectiveness, we calculated frequencies for responses regarding the effectiveness of the Council in meeting its stated objectives in the past year and in the upcoming year.

Next, the SNGA was conducted to examine the relationships among SHEC members and the opportunities for partnership development. All social network analyses were conducted using the UCInet software (Analytic Technologies, Harvard, Massachusetts). The SHEC members rated their relationship with all other members on a scale from 1 to 5 in which 1 = do not know; 2 = know only by name but would not know face; 3 = casually know as a member of SHEC; 4 = have worked together as part of a group; and 5 = have worked together personally. Network connections for nonresponding SHEC members were imputed on the basis of the responses of their fellow SHEC members. Thus, for a relationship in which only 1 person (half of the dyadic relationship) responded, we used his or her response to determine the relationship of the member who did not respond. Analyses were conducted at SHEC whole network level, at the committee level, and at the state level. Results are presented through social network maps in which a square represents a SHEC member and each line between squares represents the existence of a relationship. The size of the square represents the member's power in the SHEC network, in which actors with a larger power are presented by a larger square. Power was calculated as a weighted measure of a member's number of connections, as well as the 1 to 5 relationship rating level of those connections.

Respondents were asked to indicate what populations they worked with in regard to health. The responses were coded according to population characteristics, including race, age, income, and gender to form categories of health areas. The connections between members within these health areas were quantified by total number of connections, total number of reported 5's (personally worked with), and the lowest number of connections by a single member within each health area. If 20 people provide relationship data points for 19 others (the whole group minus themselves), there are 380 possible relationship data points (20 × 19) if we do not assume reciprocity, or 190 possible relationships if we do assume reciprocity.

To examine what organizations and affiliations are represented by SHEC members, respondents were asked to list all other affiliations they have related to the work of SHEC. We assessed SHEC partnerships by member affiliations, as well as the health disparity focus of their primary organization. We first looked at the frequencies of members presenting 4 primary health areas—race, age, income, and gender—and the existing versus potential relationships among members working in similar health areas. Next, primary organizations, as well as all affiliations, were coded to align with the sectors listed in SHEC Strategic Plan: education and research; health and human services; government; health care professionals; populations and communities; and private, civic (nonprofit and community-based), or media/communications.

## RESULTS

Out of 40 council members, 32 council members responded to the member survey. The SHEC comprises representatives from many different areas, with 31% of responding members classifying their position as working for a nonprofit organization that is primarily focused in public health, 28% of responding members classifying their position as academic working in public health, 9% of responding members as state employed working in public health, 6% as academic not working in public health, 3% as nonprofit not primarily focused on public health, and 22% in other areas, including corporate workplaces and medical services (see Table 1). No council members indicated to represent faith-based organizations or local government.

**TABLE 1.** Southeastern Health Equity Council Member Sectors

Sector	N	%
Nonprofit, public health	10	31.25
Academic, public health	9	28.13
State government, health department	3	9.38
Academic, not public health	2	6.25
Nonprofit, not public health	1	3.13
State government, not health department	1	3.13
Other	6	18.75

**Figure 1.**
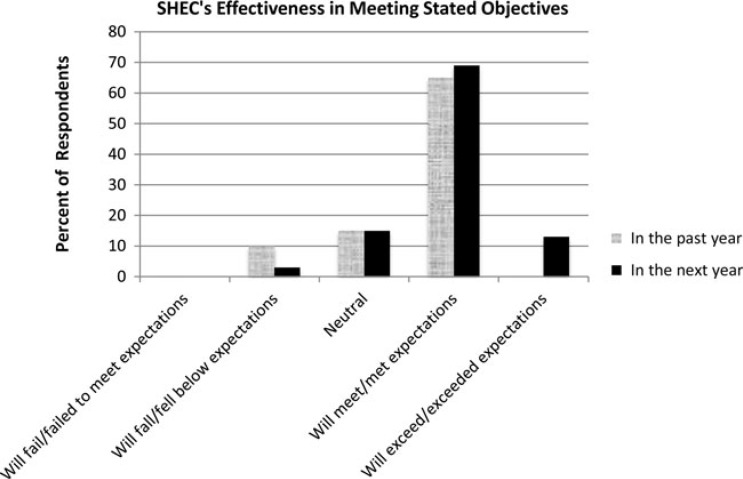
Southeastern Health Equity Council effectiveness.

Of the 5 SHEC committees, 29% of respondents were affiliated with the cultural competency committee, 23% with the social determinants of health committee, and 16% with each of the governance committee, health care access committee, and violence as a public health committee. As demonstrated in Figure 1, SHEC members are optimistic about the ability of SHEC to improve in its ability to meet its objectives within the next year.

### Social network gap analysis

We analyzed a total of 1214 relationship data points for SHEC members. The average relationship rating level was 2.67 (see Table 2 for distribution).

**TABLE 2.** Southeastern Health Equity Council Relationship Distribution

Relationship	N	%
Level 1	313	25.8
Level 2	124	11.9
Level 3	376	31.0
Level 4	235	19.4
Level 5	115	9.5

**Figure 2.**
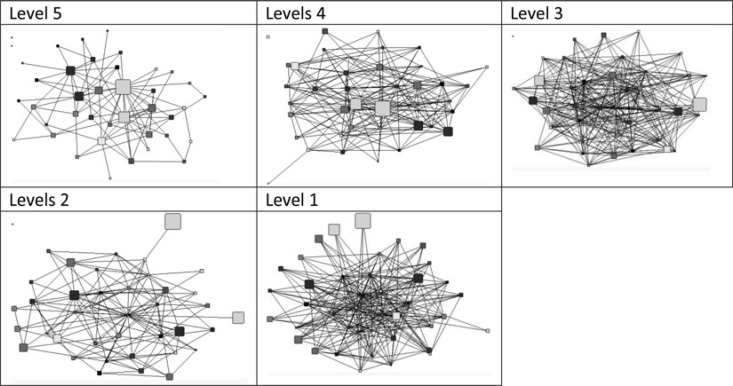
Southeastern Health Equity Council network maps by level of relationship.

In Figure 2, we map the relationships present at each of these 5 levels to visualize the prevalence of each level of partnership. Aligning with the aforementioned data frequencies, the majority of relationships (68.7%) are rated at less than 4, which indicates ample opportunities for improving collaborative activities among SHEC members. In fact, less than 10% of relationships among SHEC members are at the highest level of collaboration.

**Figure 3.**
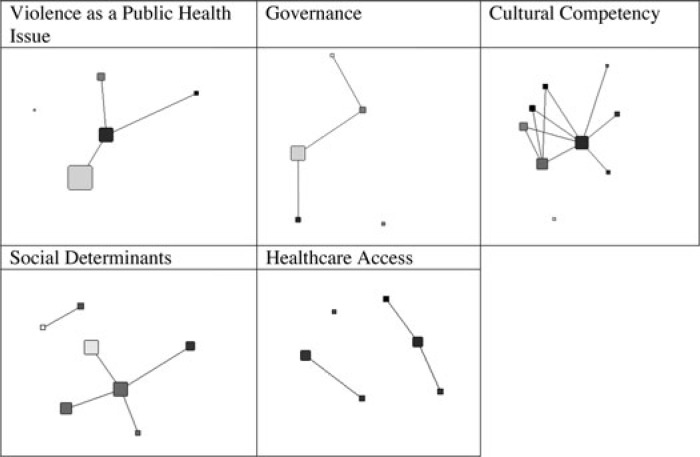
Network maps by Southeastern Health Equity Council committee.

There are differences in the composition of the networks between committees, which indicates room for improvement. The networks presented in Figure 3 represent relationships at level 3 or greater, which includes individuals who casually know each other as a member of SHEC, have worked together as part of a group, or have worked together personally. However, even when accounting for casual relationships (as well as partnerships related to public health), each committee has at least 1 SHEC member who is not connected to other members. Finally, the power positions (represented by larger squares) are distributed across the committees and the state representation (represented by colors) is well distributed across the committees.

**Figure 4.**
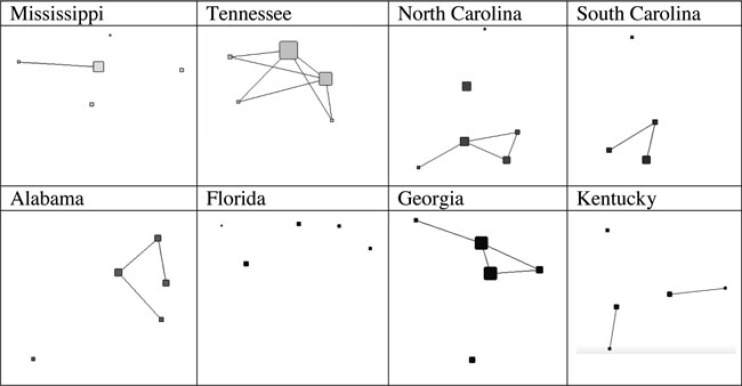
Southeastern Health Equity Council network maps by state representation.

Next, using all relationships at level 3 or above, we looked at the relationships among members by state. There is a wide range of relationships from 100% of all Tennessee SHEC members having a relationship with other Tennessee members to no Florida SHEC members having a relationship with other Florida members. We also note through visualizing the state networks that the power positions within SHEC are not equally distributed across the states that comprise the Council, which can be seen by the size of the members as represented by squares. For instance, Florida has no members holding power within the network (Figure 4).

### Affiliations analysis

The SHEC members have between 2 and 14 organizational affiliations (mean = 6.4) for a total of 199 organizations represented among SHEC members. A full list of these affiliations was compiled to develop a partnership directory that can be utilized by SHEC for both information dissemination and building strategic partnerships as opportunities arise.

Twenty Council members (50%) had affiliations in health areas involving race, which means that 380 connections could exist between members with this focus (20 respondents multiplied by the 19 members other than themselves without consideration of reciprocity). Of these, 319 (83.8%) responses indicated an existing relationship (Table 3). Of these 319 responses, just 31 (9.8%) indicated that they had personally worked together. Across the 4 groups, on average, less than 15% of all possible relationships were present.

**TABLE 3.** Southeastern Health Equity Council Health Area Connections

Health Area	N^a^	Existing Relationships (Relationship Levels = 2-5), %	“Personally Worked Together” (Relationship Rating Level = 5), %	Minimum Connections (the Percentage of Group Members That All Members Have at Least a “2” Connection With)
Race	20	83.8	9.8	55.0
Age	14	72.5	16.6	28.6
Income	12	68.9	16.5	33.3
Gender	8	57.1	12.5	37.5

^a^Some Council members listed more than 1 health area and as a result, the N value does not sum to 32.

In addition to assessing affiliations for relationship opportunities, we coded affiliations by sector to develop a better understanding of member representation distribution among the sectors identified in the SHEC Strategic Plan: education and research; health and human services; government; health care professionals; populations and communities; and private, civic (nonprofit and community based), or media/communications. This information could then be used to recruit new members in these areas as vacancies arise. The results indicate that the areas of health care and media/communications are underrepresented (see Table 4). For instance, 16 SHEC members reported a primary organization that was academic/education related.

**TABLE 4.** Organizations and Affiliations of Members by Sector

	Primary Organization	Secondary Organization	First Affiliation	Second Affiliation	Third + Affiliations	Total Affiliations
Education and research	16	5	6	3	9	39
Health and human services	5	4	4	1	7	21
Government	3	3	1	0	7	14
Health care professionals	2	2	0	0	0	4
Populations and communities	2	2	3	1	4	12
Private	2	2	1	4	0	9
Civic, nonprofit, and community-based	1	13	15	16	58	102
Media/communications	0	0	0	0	0	0

### Recommendations drawn from SHEC's SNGA

The SNGA revealed many strengths and weaknesses in the case of SHEC. The strengths consisted of the presence of a strong central actor among the majority of committees, showing leadership and relationship impact. In addition, as a whole, most members know each other by face and are working together for common public health goals. However, the SNGA also revealed many opportunities for both improving these relationships and strengthening SHEC. It was noted that in 1 state, in particular, Florida, members were not working together at all, and there was an opportunity to bridge 10 absent relationships. We also note, however, that at the time of the survey, Florida members were new council members (ie, in their first year serving the Council) and 2 of the Tennessee members, where the network was the strongest, had the longest tenure on the Council. We also noted the absence of many relationships within committees. Based on the results of the SNGA, recommendations were made to SHEC in 2 areas: (1) collaboration and partnerships and (2) filling strategic gaps within the Council. Within these areas, this study yields 8 specific recommendations, which are marked within parentheses throughout this section.

First, in regard to collaboration and partnerships, there is a significant gap in the number of, and strength of, relationships between incoming and returning members (those with 2 or more years on the Council) of SHEC. For instance, much of the gap between the Florida and Tennessee network structures can be attributed to the tenure of the members from those states, with the former comprising new members and the latter comprising returning members. Based on this observation, we recommend an (1) *onboarding mentorship program in which returning members are paired with new members to provide guidance in efforts related to the mission of SHEC*. Mentors can be identified on the basis of common state association, common health area, or common committee within SHEC, all of which can be identified through the tool introduced in this study.

The SNGA of the SHEC committees revealed that not all members of each committee are working as part of that committee and that there are distinct differences in the level of collaboration when comparing committees. Based on the observation of differences across committees, we recommended that (2) *each committee work to include all members of their committee in current projects and programs* and (3) *the success of the committees with higher degrees of collaboration be documented and replicated in the committees with lower levels of collaboration*. Here, we also note that the Violence as a Public Health Issue committee was formed just 4 months prior to the survey, which may partially explain the differences for this committee.

We find that state collaboration is much more limited than committee collaboration. With the exception of Tennessee, Council members do not know all other members in their state. Based on this observation, we have 3 recommendations: (4) *leadership positions within SHEC should be sensitive to states represented to ensure that actor power is distributed among the states*, (5) *states should coordinate efforts to communicate around health issues, events, and programs relevant to their individual states*, and (6) *a state leadership designation should be made for an individual within each state responsible for coordinating that state's Council members*.

Second, the SHEC SNGA provides information useful for strategically filling gaps within the SHEC as membership openings become available. No Council members represent faith-based organizations or local government, and there is limited representation from media/communication. Given the role that these stakeholders play in health in the Southeast, we recommend representation from these communities on SHEC or as advisors to SHEC. We find that there are 200 organizations represented on the Council (inclusive of SHEC), which presents a strength of the Council in terms of constituency and dissemination. However, this strength is not fully achieved when Council members are not active within SHEC. Based on this observation, we recommend that (6) *a Partnership Directory, which for SHEC was developed using the survey and SNGA presented within this article, be utilized to identify the affiliations of SHEC members and to utilize these partnerships strategically for both input (expert knowledge of specific health areas and regions) and output (dissemination/communication of efforts of SHEC).*

According to the data, the connection among SHEC members within each of the health area demographic groups (ie, race, age, income, and gender) was relatively strong. However, the number of members who had reported working personally with these group-based potential connections was low. Therefore, we recommend (7) *an intervention that targets deficiencies in collaboration among members working on similar issues in public health*. The SNGA can be further utilized to identify a member from each group with a high number of connections—a high network power. He or she would be responsible for identifying areas of collaboration between specific members. This would increase the amount of relationships in the network that could report working personally with each other. Similarly, within the organizations and affiliations data, the lack of media/communications respondents shows a need for expertise in this area. We recommend (8) *a search be conducted to identify personnel in this sector who could fill this gap and contribute to SHEC.*

As a final recommendation to SHEC, this study should be reproduced annually following the implementation of the aforementioned recommendations to assess progress in SHEC social networks. The data provided herein can serve as a baseline evaluation for charting growth in the areas of collaborations and partnerships and in strategic gaps in partnerships. We also recommend that this study be replicated in other Regional Health Equity Council regions to allow for each of the regions to identify their own strengths and weaknesses in collaborations, partnerships, and representation. This will also allow the Office of Minority Health and the NPA to identify regions, committees, and states that exhibit exemplary collaboration and for these to be studied and replicated. Finally, these data can be used to assess the relationship between strong collaboration and effectiveness in work toward eliminating health disparities in the United States.

## DISCUSSION

In this article, we have used SNGA methods to measure partnerships with the SHEC as a whole, within committees, state networks, and among members working on similar public health issues. In this case study, partners within SHEC vary in their network connectivity. Variability results from differences in resources, availability, priorities, and level of integration into the network.^30^ Despite recognition of the causes of differences in involvement among members of SHEC, the lack of connectivity among members influences the ultimate effectiveness of the Council.^30^–^34^ By employing SNGA, we have highlighted systems-level characteristics of the network that might be limiting the effectiveness of SHEC.

Although many health organizations have begun to pursue collaborative approaches for addressing community-level health issues, there remain limited tools for evaluating these approaches.^5^ This research seeks to add to the tools and body of literature on partnership evaluation. By discussing the SNGA methods used, data results, and recommendations made in the case of SHEC, we have demonstrated how the methods employed herein can be used to assess gaps in partnerships. More specifically, this research advances the literature on SNGA through presenting the case study of SHEC, in which we empirically tested and documented partnerships. The SHEC's stated purpose—“to build collaboration and partnerships to achieve health equity in the Southeast region of the US”—is regionally specific but similar to many other councils working to improve community health, minority health, and health disparities. Evaluating the networks formed as part of these collaborations is imperative to the advancement of these efforts. As demonstrated here, SNGA has enhanced SHEC's ability to meet its mission of informing policy and reducing health disparities in the American southeast. More broadly, this case study demonstrated how community organizations can increase accountability around collaborative efforts to address health disparities. For a gap analysis perspective, this has been accomplished by assessing the network as it exists to make recommendations for improving the network.

Just as SNGA draws from social network analysis and theories, as well as gap analysis, it contributes to evaluation studies and methods in these areas. It also contributes to the literature on public health evaluation. While 8 specific recommendations were developed for SHEC using these analysis methods, the recommendations would vary on the basis of the case examined. However, the tools can be applied and adapted across cases on public health collaboration. Given the common need to measure the formation, structure, and progression of relationships to improve council structure,^15^–^17^ the SNGA holds many opportunities for understanding the relational processes at play when groups form to address public health issues. It also holds opportunities for understanding how the network structure may promote or inhibit the success of the council.^5^,^14^ The recommendations that were provided to the SHEC demonstrate the value of SNGA in using network gaps to design initiatives to promote the effectiveness of future efforts.^18^–^20^

Several factors limited this study. Although the member survey had a good response rate,^35^–^37^ missing data may have affected network measures. Specifically, this would result in the overstatement of gaps or the understatement of cohesiveness. A second limitation of this study is that by the case study design, the network data presented herein cannot be generalized to other networks. The study also has several strengths and significant contributions. First, SNGA has been introduced and exhibited as an innovative method for providing metrics of network collaboration. This research has provided a network perspective for conducting gap analyses through the case study of SHEC and can provide a model for network assessments for other evaluators and researchers.

## CONCLUSION

The complexity of health disparities has resulted in specialization among individuals working in public health. In response, public health efforts often focus on coalition building to bring together collaboration across multiple partners in a culturally sensitive and contextual way. Although public health partnerships and collaboration bring together diverse individuals with unique contributions to help meet the challenges of health disparities, there remains dearth of literature providing tools for council evaluation. Through a presentation of the SHEC case study, we have presented the methods by which SNGA can be conducted to evaluate public health networks for the purpose of improving their effectiveness. Applying SNGA to evaluate the SHEC produced 8 recommendations for improving the council network. Future research is recommended to assess the effectiveness of these recommendations if enacted by the SHEC and apply the SNGA across more and diverse public health councils.
